# Investigation of the Wetting Properties of Thalassemia Patients’ Blood Samples on Grade 5 Titanium Implant Surfaces: A Pilot Study

**DOI:** 10.3390/biomimetics8010025

**Published:** 2023-01-07

**Authors:** Ali Temelci, Hasan Güney Yılmaz, Gürkan Ünsal, Lokman Onur Uyanik, Dilek Yazman, Aysa Ayali, Giuseppe Minervini

**Affiliations:** 1 Department of Oral and Maxillofacial Surgery, Faculty of Dentistry, Near East University, Nicosia 99138, Cyprus; 2 Department of Periodontology, Faculty of Dentistry, Near East University, Nicosia 99138, Cyprus; 3 Department of Dentomaxillofacial Radiology, Faculty of Dentistry, Near East University, Nicosia 99138, Cyprus; 4 Center of Thalassemia, Dr. Burhan Nalbantoğlu State Hospital, Nicosia 99010, Cyprus; 5 Department of Oral and Maxillofacial Surgery, Faculty of Dentistry, European University of Lefke, Nicosia 99728, Cyprus; 6 Multidisciplinary Department of Medical-Surgical and Dental Specialties, University of Campania Luigi Vanvitelli, 81100 Caserta, Italy

**Keywords:** prosthesis, biomimetics, thalassemia, dental implants, wettability, osseointegration

## Abstract

Background and Objectives: Beta-thalassemia (BT) has a high prevalence in Mediterranean, Southeast Asian, and African countries. Studies stated that thalassemia is an endemic disease that causes significant health problems in Cyprus. This study aimed to measure the contact angle between the implant and blood samples from BT major patients and healthy individuals to compare the contact angles and wettability of Grade 5 titanium implant surfaces. Materials and Methods: Grade 5 titanium discs that were 10 mm in diameter were used since they mimic the surface of dental implants. Following receiving informed consent, blood samples were taken from the patients’ index fingers in each group with lancet needles and a photo of the contact angle between the blood samples and the titanium surface was taken; the collected blood was transferred to a titanium disc with a medical pipette. ImageJ software with a specific contact angle plugin was used for the contact angle measurements. Results: Theta-mean, theta-circular, and theta-ellipse values were compared between all groups, and no significant difference was found (*p* > 0.05). Conclusions: In this study, it was hypothesized that the patients’ rheological property of decreased deformability would affect the wettability of implant surfaces in vitro; however, no such finding was reached in this study. Since in-depth studies associated with dental implant success in BTM patients are absent in the literature and Cyprus is one of the Mediterranean countries with a high prevalence of BTM, this study was conducted to enrich the literature. While some systemic diseases may affect the contact angle between the implant surface and blood, it can be concluded that this condition was not present for BTM patients in our study. Last but not least, we emphasize that this experiment was done on a single surface type and the results can be totally different when using other surface types.

## 1. Introduction

Thalassemia is a cause of hypochromic microcytic anemia, which arises due to the damaged synthesis of the hemoglobin’s globin chain. β-thalassemia (BT), which is considered one of the most frequent hereditary disorders, refers to a reduced β-globin chain of hemoglobin due to a mutation of the β-globin gene [1,2]. BT syndromes result in anemia, reduced hemoglobin in red blood cells (RBC), decreased RBC production, and increased platelet levels. Most thalassemia is inherited as recessive traits and three classifications of BT are present: BT major (Cooley’s Anemia), BT intermedia, and BT minor. While the hematological and clinical symptoms are more dangerous in BT major (BTM), insignificant findings that may not even require treatment are generally associated with BT minor [3,4]. 

As dental implants are placed inside the jaws, numerous biological and chemical interactions are initiated between the periodontal tissues and the implant surface. The first contact of the implant surface within the bone is with the blood, which carries chemokines, cytokines, and growth factors that stimulate new bone formation in the osteotomy area [5,6,7]. Unless the blood sufficiently wets the surface of the implant, this can lead to poor healing or delays in healing; hence, the contact with blood directly affects the osseointegration, and wettability is one of the non-clinical criteria for the survival of the implant in the mouth [5,8,9].

Due to its biological and mechanical characteristics, pure titanium and its alloys are often employed in biomedical applications. Surface alterations are required to promote osseointegration at bone–implant interfaces, even if titanium’s mechanical characteristics allow for acceptable responses to dynamic stresses. There are numerous different ways to modify a surface, such as sandblasting, acid etching, or coating. There is currently no agreement on the ideal surface characteristic values for a successful dental implant despite the fact that numerous studies conducted assessments of surface characteristics of dental implants using comparable procedures but with various measurement parameters. Surface roughness, among many other surface properties, is one of the most crucial elements for dental implants. Because of this, the experiment in this investigation used an implant disc type that was approved by accredited laboratories [10,11]. In addition to surface characteristics, the bulk physical parameters of an implant material also affect biocompatibility. Ti and Ti alloys are particularly common biological implant materials in biomedical engineering and industry due to their bulk physical characteristics and strength-to-weight ratio approaching those of natural bone. The titanium surface’s physical characteristics can be changed to improve surface biocompatibility. To create regulated nano/microscale roughness on the titanium implant surfaces, the chemical mechanical polishing (CMP) approach was presented as a new alternative [11]. Current biomaterials research emphasizes the role that nanoscale roughness plays in enhancing biocompatibility on metallic implant surfaces. By using this innovative technique on implants, the out-diffusion of titanium and other metallic impurities from prostheses in contact with bodily fluids and tissue is to be considerably reduced, while at the same time, the surface’s mechanical, chemical, and biological properties are improved. On Ti and Ti alloy samples, the CMP process permits the creation of a thicker and denser self-protective native oxide while also inducing a regulated surface roughness [11,12,13,14,15,16,17,18,19].

In order to increase the wettability of implant surfaces, surface modifications have been made with many different methods. However, the surface wettability may not only depend on the surface properties but may also vary depending on the properties of the liquid that wets the surface [6]. Based on the studies conducted to date, which concentrated on the evaluation of the wettability of implant surfaces, there was no study that investigated the role of blood properties in BT patients in the wetting of implant surfaces. Many different surface modifications, such as sandblasting, acid etching, calcium phosphate coating, and combinations of these, were created and tried on the implant surfaces to ensure faster and more effective osseointegration to increase cell retention, surface tension, and wettability on the implant surface. After becoming aware of the role of a certain microroughness for improved cellular contact and osseointegration of dental titanium implants, the likewise important role of surface energy and wettability was increasingly strengthened [9].

BT has a high prevalence in the Mediterranean, Southeast Asian, and African countries, and due to migrations, Northern European countries had a sharp increase in recent years. BTM patients generally require a medical intervention between 24 months and 6 years, as RBC transfusions are essential for their survival. Most of the patients without a prenatal diagnosis in developed countries get their initial diagnosis during that time, and both iron chelation therapy and a transfusion program are set [20]. Patients who cannot get blood transfusions suffer from bone deformities, abnormal growth, and chronic anemia [21,22]. BT patients can develop iron overload that may damage their hearts and lungs. The most common causes of mortality and morbidity in BTM patients are damaged pulmonary and cardiac functions [23].

As a result of the ineffective process of erythropoiesis, erythroid marrows are expanded and hypertrophic in BTM patients. Hence, maxillary protrusion, malocclusion, anterior open bite, and bossing of the zygomatic and frontal bones are seen. Moreover, studies showed that BTM patients are at high risk for periodontal diseases and tooth caries, which can lead to early and multiple teeth loss [24,25,26]. In order to provide masticatory functions, dental implant treatments can be used since titanium and titanium alloy implants have near-perfect biocompatibility, which can be modified even in challenging cases [7,27,28,29].

The osseointegration process in dental implants includes the formation of blood clots for wound healing and the immigration of granulocytes and monocytes to the wound to clean the area and produce growth factors for angiogenesis, which then follows stimulation of fibroblasts for producing extracellular matrix, ending up as bone regeneration [30,31]. BT patients suffer from late wound healing, which is mainly attributed to low hemoglobin that reduces tissue oxygenation, decreased deformability, and increased agreeability of erythrocytes attributed to high platelet levels that negatively affect perfusion [30,31,32,33,34]. Hence, due to these rheological differences between a healthy individual and a BTM patient, we hypothesized that a dental implant treatment will be less successful, which is reflected in the measurement of wetting of the implant surfaces. In order to test our hypothesis, this pilot study aimed to measure the contact angle between the implant and blood samples from BTM patients, BT carriers, and healthy individuals to evaluate and compare the contact angles and wettability of Grade 5 titanium implant surfaces to guide future clinical studies. 

## 2. Materials and Methods

### 2.1. Ethical Considerations

The study protocol was approved by the Scientific Research Ethics Committee (Project number NEU/2021/89-1319, meeting number: 2021/89) on 25 March 2021 and by the Turkish Republic of Northern Cyprus Ministry of Health Scientific Research Ethics Committee (file number: 2021/46-21) on 11 October 2021. The study was performed in accordance with the tenets of the 1964 Helsinki Declaration and its later amendments. An informed consent form was obtained from each participant. 

### 2.2. Patient Selection and Study Materials

This study was conducted with 42 BTM patients, 42 beta-thalassemia carriers, and 42 healthy individuals in the Dr. Burhan Nalbantoğlu Center of Thalassemia. 

For the experimental setting, a schematic with multiple compartments was set. The setup consisted of the following:

- High-resolution camera with a macro lens - Nikon D750, Nikon 105 mm Macro Lens (Nikon, Tokyo, Japan)
- Camera tripod for the stabilization of the camera
- Fixed medical pipette to drop the blood sample on the titanium surface
- Base to fix the Grade 5 titanium discs
- An illuminating system
- Titanium implant discs (see Section 2.3).

### 2.3. Features of the Titanium Implant Discs

The report (report number: 72602115-6020402-1580) is the result of the study carried out by the Istanbul Technical University Manufacturing Technology Application and Research Center to determine whether our samples had a “Resorbable Blast Media (RBM) Surface, Resorbable Blast Texturing Surface (RBT) and Soluble Blast Media (SBM) Surface” and to determine the surface roughness values. An optical profilometer was used (AXIO CSM 700, Zeiss, MN, USA) to evaluate the surfaces of the titanium implant discs, and the report was as follows: “In the technical examinations and analyzes, it was determined that it was sanded with structures in the form of tricalcium phosphate and hydroxyapatite, therefore it had RBM, RBT and SBM surfaces. The surface roughness analysis results made with a profilometer device and a 12.5 µm tip on the same samples were 1.687 µm, 1.677 µm and 1.727 µm for 3 different samples, respectively. found in microns.”

The summary of the implant disc’s features was as follows:

- Grade 5 titanium discs (Mode Implant) with 10 mm diameter were used for all patients’ blood samples for the contact angle measurements;
- Type: Grade 5 (Ti6Al4V);
- Surface Features: resorbable blast media surface, resorbable blast texturing surface, and soluble blast media surface;
- Brand: Mode Implant;
- Diameter: 10 mm;
- Surface roughness: 1.697 µm;
- Surface sanding materials: tricalcium phosphate and hydroxyapatite. Biphasic calcium phosphate micro-blast with 65% HA content was used to sand the implant surface homogeneously at 1.4–1.8 µm with ROBOT technology.

**Figure 1:**
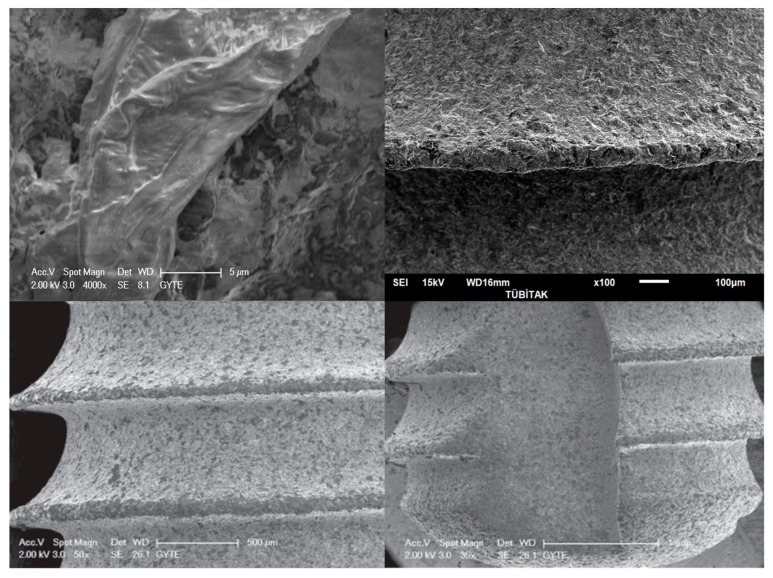
Scanning electron microscope images of the Grade V implant discs that were used in the present study. The magnifications of the images are 4000× (upper left), 100× (upper right), 50× (lower left), and 35× (lower right).

High- and low-magnification scanning electron microscope images of the titanium implant surfaces are shown in Figure 1 with multiple magnification rates.

### 2.4. Blood Sampling 

Following giving informed consent, the donor’s index finger was pierced with a lancing device with push button ejection to collect a blood sample. As the blood that emerges following the piercing process contains more tissue fluid and less hemoglobin, it was wiped with a sterile gauze cloth. Then, light pressure was applied to the donor’s index finger and a medical pipette was used to collect the proper sample, which was transferred to the implant disc surface immediately. 

In order to eliminate any bias, as the patients with BTM would have similar blood features and wettability properties to healthy individuals following their blood transfusions, the samples were collected a day before their transfusion process. 

### 2.5. Imaging of Blood Samples

**Figure 2:**
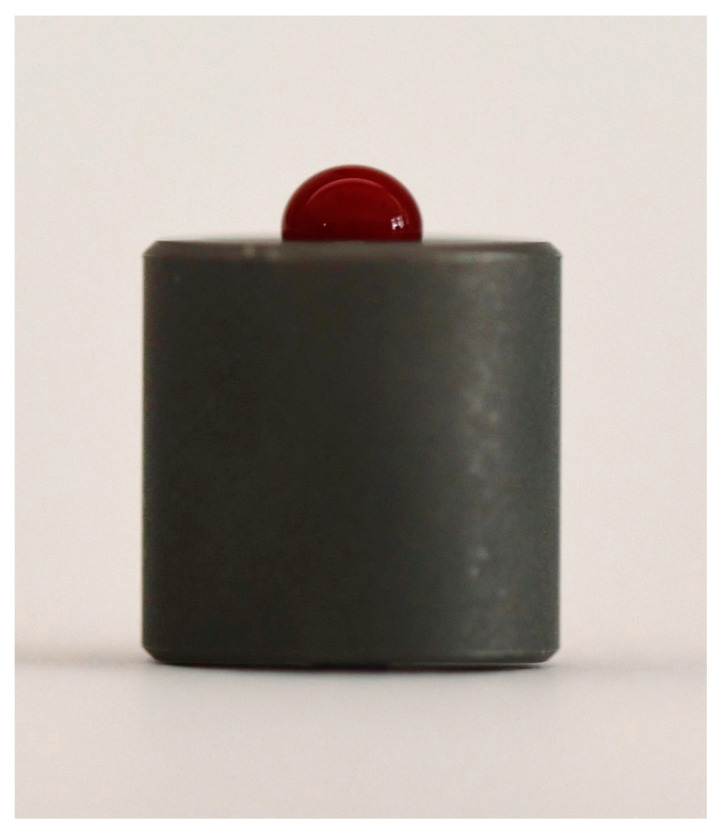
Following the transfer of the collected blood with a medical pipette on the titanium disc, photos were taken without modifying the settings for all samples.

In order to take a photo of the contact angle between the blood samples and the titanium surface, the collected blood (0.05 mL) was transferred to the titanium disc with a medical pipette, and photos were taken 5 s after the procedure (Figure 2). No anticoagulants were used since the transfer time was short. A single disc was used for each patient, and the photos were transferred to a computer with an SD card to measure the contact angles. 

### 2.6. Isotonic and Hypotonic Water 

As only a single type of implant disc could be obtained for this study, in order to compare the blood samples with a reference, the same experiment was performed with distilled and isotonic water.

### 2.7. Contact Angle Measurements

**Figure 3:**
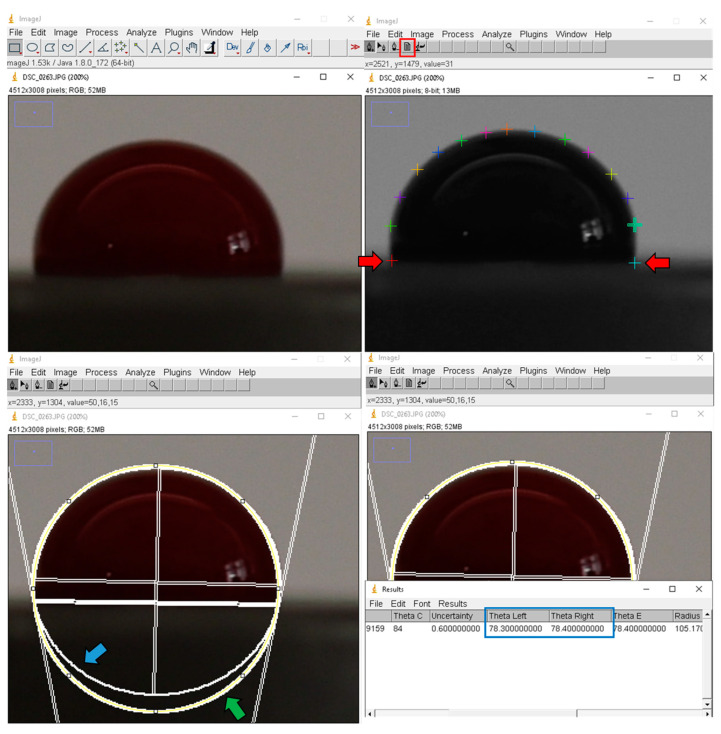
Contact angle measurement with ImageJ’s Contact Angle plugin. Following the manual selection of the first 2 points (red arrows) to determine the baseline, the periphery of the blood sample was marked to calculate the contact angle between the surface and blood. It is important to point out that an ellipse best-fit analysis poses itself between the circle best fit (green arrow) and an absolute best-fit function. Sometimes the drop is not perfectly symmetric or there is a slight effect due to gravity. In this case, an almost-circle (blue arrow) analysis can provide useful results.

Contact angle measurement, also known as the θ (theta) degree, is a quantitative measure of the wetting of a solid by a liquid. The angle created by a liquid at the intersection of a liquid, gas, and solid at a three-phase boundary is known as the contact angle [35]. ImageJ software v1.53t (NIH, Bethesda, MD, USA) with a specific contact angle plugin (Contact Angle plugin for ImageJ) was used for the contact angle measurements. This plugin, which was based on another plugin called “Pointpicker” written by Marco Brugnara, calculates the contact angle between a drop and a flat surface with an approximation [2atan (2 h/L)] and an ellipse approximation. This plugin requires a manual baseline determination by choosing the 2 initial points, as shown in Figure 3. 

The guide of the plug-in suggests the use of high-resolution and well-defined pictures, as the best-fit analysis automatically detects the profile of the droplet; thus, a high-resolution camera with a macro lens (Nikon D750, Nikon 105 mm Macro Lens) was used in our study. It was also stated in the guide that “It is important to point out that an ellipse best-fit analysis poses itself between the circle best fit and an absolute best-bit function. Sometimes can happen that the drop is not perfectly symmetric, or that there is a slight effect of the gravity. In this case an almost-circle analysis can provide useful results.” https://imagej.nih.gov/ij/plugins/contact-angle.html (accessed on 12 March 2022).

Four different preferences are present for the contact angle measurement: manual point selection, circle best-fit measurement, ellipse best-fit measurement, and application of both circle best-fit and ellipse best-fit measurements. As the most reliable way of determining the region of interest is manual point selection, we determined the profile of the droplet manually by placing more points along the droplet’s edge. 

To avoid any user-related errors, all measurements were repeated 5 times for both the left and right sides, and the arithmetic means in degrees were calculated. Then, the arithmetic means of the right and left contact angles were used for the statistical analysis since those two angles may demonstrate minor differences. As the software can fail when a non-straight triphase line is provided, all measurements were repeated in cases with an uncertainty value higher than 1.

### 2.8. Statistical Analysis

Contact angle data were analyzed with SPSS software v22.0. The Kolmogorov-Smirnov test was used to test the conformity of the data to the normal distribution, and for the continuous variables, the median, minimum, maximum values, and mean ± standard deviation are given. Levene’s test was used to evaluate the homogeneity of the variances between the groups, Dunnett’s T3 test was used for statistically significant measurements as a post hoc test, and the Welch ANOVA test was used to compare the heterogeneous measurements of the variance between the groups. Measurements that did not comply with the groups’ normal distribution were compared with the Kruskal–Wallis test. The Mann–Whitney U test was used as the post hoc test of the statistically significant measurements. A Bonferroni correction was used to interpret the results. A Friedman test was used for the variables that did not have a normal distribution among the group. A Bonferroni correction was used to interpret the *p*-values, and cases with *p* < 0.05 were considered significant.

## 3. Results

### 3.1. Patient Demographic Data

A total of 126 patients were enrolled in the present study (Table 1). The mean ages were 35.85 (min 21, max 66) for the healthy group, 37.3 (min 22, max 64) for the carrier group, and 39.7 (min 20, max 55) for the BTM group. Twenty-two females and 20 males were enrolled in the healthy and BTM group, while 23 females and 19 males were enrolled in the carrier group.

### 3.2. Blood Sample Features of the Participants

Blood sample features of the BTM patients and others are noted in Table 2. Mean values of RBC (million cells/mcL), HGB (gr/dL), and PLT (Thousand Platelets/mcL) values were 3.76, 9.91, and 525 for the BTM male patients and 3.79, 10.09, and 470 for the BTM female patients, respectively. The mean values of RBC, HGB, and PLT were 5.52, 15.3, and 324 for healthy males and BT carrier males and 4.83, 13.5, and 317 for healthy females and BT carrier females, respectively.

The statistical analysis results of the blood sample features between the groups are given in Table 3. There was a significant difference in all parameters between BTM patients and non-BTM patients (*p* < 0.05) and there were significant differences in RBC and HGB between the non-BTM females and non-BTM males. No significant difference was found in all parameters between BTM males and BTM females, and no significant difference was found in PLT between non-BTM males and non-BTM females.

### 3.3. Angular Measurements

All contact angle measurements were recorded in this study as theta-mean, theta-circular, and theta-ellipse. The mean contact angles (theta-mean) values were 89.98 (min 69.15, max 125.45) for the BTM group, 87.59 (min 68.4, max 117.1) for the healthy group, and 85.79 (min 67.95, max 105.1) for the carrier group. The mean theta-circular values were 93.85 (min 75.7, max 127.4) for the BTM group, 89.89 (min 70.1, max 111.4) for the healthy group, and 88.02 (min 76.9, max 110.5) for the carrier group. The mean theta-ellipse values were 89.98 (min 69.2, max 125.4) for the BTM group, 87.6 (min 68.4, max 117.1) for the healthy group, and 85.77 (min 67.9, max 105.1) for the carrier group (Table 4).

The mean theta-mean values for the distilled water and isotonic water were 103.03 and 102.14, respectively.

### 3.4. Uncertainty Results

Uncertainties in the blood droplet shape adversely affected the contact angle studies. An uncertainty value of around 1 μm in the baseline location or height of a droplet can lead to uncertainties as large as 10° in the contact angle measurements. In order to eliminate any falsely measured contact angles, we repeated the contact angle measurement for cases that had an uncertainty value higher than 1. The mean uncertainty values were 0.52 for the BTM group (min 0.2, max 1), 0.61 for the healthy group (min 0.2, max 1), and 0.65 for the carrier group (min 0.3, max 1). 

### 3.5. Statistical Analysis of the Contact Angles

The theta-mean, theta-circular, and theta-ellipse values were compared between all groups, and no significant difference was found between the groups, which suggested that blood samples of all groups had similar wettability properties in this in vitro study (*p* > 0.05) (Table 5).

## 4. Discussion

Dental implants are highly successful in patients without systemic diseases and with good oral hygiene; however, further studies should be conducted to see their performances in compromised patients with systemic diseases [9,22,36,37]. This study was the first to evaluate the effects of BTM on Grade 5 titanium dental implant discs. The measurements were done with non-clotted blood drops to examine the real-time first interaction between the disc and the blood, where we avoided collapsing the blood sample’s peak. As no substrate was added to the blood samples, our findings are more significant than the ones with an addition [8].

The significant differences between the non-BTM females and non-BTM males in terms of the RBC and HGB parameters were expected, as the reference values of each of these two groups were different in healthy individuals; as the PLT reference values were the same in both females and males, it was also expected not to have a significant difference between the non-BTM females and non-BTM males [1,2]. The statistically significant differences in RBC, HGB, and PLT values between BTM patients and non-BTM participants allowed us to conduct this experiment with a dioristic approach. Although the RBC and HGB values were significantly different in healthy individuals, these values were not significantly different between BTM males and BTM females; hence, according to the results of this study, it can be concluded that having BTM was more influential on the blood features than gender. This study hypothesized that blood samples of BTM patients would have significantly different contact angles than those of healthy patients due to these rheological differences between a healthy individual and a BTM patient; however, our study found no significant differences between the BTM, carrier, and healthy groups.

PubMed Database was searched for any implant procedure for a patient with Thalassemia with the following query box “((Thalassemia) AND (implant)) AND (jaw)” and three studies were found [3,38,39]. Misch et al. reported the first dental implant case report of a BT patient in 1998 who had characteristic BTM clinical and radiographic findings, such as generalized osteolytic lesions at the alveolar bone, a coarse and decreased trabecular pattern, and thinning of the cortical plates. Instead of conventional titanium implants, they used hydroxyapatite-coated implants. Even though the patient’s bone quality was rated as a type IV bone, uneventful healing was achieved and no peri-implantitis was present during the regular 3-month recalls. Due to the maxillary bone marrow hyperplasia, an increased bone volume was seen in their case. In order to address these given clinical features, they modified the implant biomaterial, surgical approach, and prosthetic design. They concluded that the surgical technique, implant biomaterial, and prosthetic rehabilitation were modified in this case to address these conditions [3]. Pektaş et al. reported the first case report of a segmental maxillary osteotomy with implant application in BTM patients, and they described the complications seen during that procedure. Primary stability problems and overbleeding were underlined, and they suggested further studies to assess the implant survival rate in BTM patients [38]. Ören et al. reported a 39-year-old BTM patient who was referred to them with severe facial deformities and periodontal problems. They extracted all the patient’s teeth and applied custom-made sub-periosteal implants. They reported no uneventful complications at the 3-year follow-up [39].

This study was carried out to achieve a significant breakthrough in the literature as a pilot study, as there are no comprehensive studies related to dental implant success in BTM patients, and Cyprus is one of the Mediterranean countries with a high prevalence of BTM [40,41,42]. Modell’s report in 1979 [41] and Kolnagou’s article in 2009 [42] concluded that thalassemia is an endemic disease that causes a significant health problem in Cyprus. Modell reported that 15% of the Turkish Cypriot population are thalassemia carriers, and Kolnagou reported that one in six people is a heterozygote thalassemia carrier. More importantly, 1 in 1000 people is a homozygote thalassemia major patient in Cyprus. Thanks to the legislation mentioned by Modell et al., severe economic, psychological, and social problems were alleviated. Were it not for the thalassemia policies, most patients would die before reaching 7 years old due to the absence of blood transfusions. Nowadays, patients dependent on blood transfusions are getting treatment in major cities in Cyprus [40,41,42]. As discussed by Tillmann et al., in heterozygotes, decreased deformability/flexibility is a well-established property of beta-thalassemia patients, primarily due to microcytosis. At the same time, in homozygotes, it was attributed to severe cell shape alterations and inclusion bodies in the splenectomized patients. Their study also found that the deformability of erythrocytes in frequently transfused patients showed a substantial decrease, while heterozygotes’ erythrocytes showed only a moderate decrease in deformability. One might assume that BTM patients’ blood features would have similar wettability properties to healthy patients if they have frequent blood transfusions; hence, the blood samples from those patients were collected shortly before their transfusion procedure [43].

Although cytotoxic effects of vanadium were shown by Dalal et al. and Zwolak et al., Zhang and Chen compared the strength of Grade 4 (pure titanium) and Grade 5 (titanium, vanadium, and aluminum alloy) implants and reported that the strengths of those implants were 550 MPa and 930 MPa, respectively [44,45,46]. It was decided to use Grade 5 implant surface discs in this study, as Willis et al. stated that stronger dental implants that are shorter and/or narrower can also be placed without an increased implant fracture risk in patients with narrow alveolar ridges or a need for vertical alveolar bone grafting [47,48].

The resorbable blast media (RBM) surface treatment is intended to create a rougher implant surface without leaving behind any implanted blast debris, which makes it superior in terms of residual particles. As higher roughness for the surface increases the osseointegration rate, this parameter is essential to increase the bone-to-implant contact and the tissue growth at the peri-implant structures without any uneventful post-operative conditions. RBM is primarily responsible for roughened implant surfaces and boosts retention power. The topography of RBM influences the attachment and the growth of the new bone cells in a more stable and faster way. Calcium phosphate is the substance utilized in the RBM procedure. Since calcium phosphate is a biocompatible material that has high resorption rates, no strong acid application is needed to remove the residuals of the material. Even if the residuals of calcium phosphate may not be removed, as these residuals are entirely biocompatible, they can promote earlier bone in-growth and provide greater implant stability during the first critical weeks after dental implant placement. The RBM method, which consists of hydroxyapatite (HA) blasting and soft etching of the surface of the dental implants, is a widely utilized substitute for the bioincompatible alumina blasting techniques. Additionally, the other benefit of this surface type is soft chemical therapy. These benefits guarantee a high level of surface cleanliness without any possibility of alumina contamination or surface damage [49,50,51]

The soluble blast media (SBM) surface is manufactured with a similar approach to the RBM technique; however, in SBM surfaces following the HA crystals application to roughen the disc surface, the surface is cleaned with a 20% acidic solution. SBM surfaces are etched less by the acids (compared with the 40% nitric acid etching), which is beneficial for osseointegration, and have rougher surfaces than RBM surfaces owing to the etching procedures [16,49,51,52,53].

In a study that was conducted by Mekayarajjananonth et al., it was found that distilled water had a lower contact angle (and a better wettability) on Grade 5 implant surfaces with a calcite hydroxyapatite plasma spray coating than Grade 1 implant surfaces [52]. On the other hand, when the test was conducted with glycerol instead of distilled water, they reported that there was no significant difference in contact angle; hence, it can be concluded that the liquid that was tested on the surfaces was also as important as the surface characteristics. They also stated that multiple factors affected the contact angle, such as the surface preparation method, structural features of different implant materials (such as Grade 1 and Grade 5 implants), surface roughness, and microbial contamination. As proof of the importance of the preparation method, they also reported that while the Ti6Al4V calcite hydroxyapatite plasma spray coating surface had the best wettability, the Ti6Al4V MP-1^®^ hydroxyapatite plasma spray coating surface had a worse wettability (according to their contact angles) [54]. 

Effects of biomaterial relations on surface hydrophilicity were reviewed by Spijker et al. According to their study, the activation of the adhesion and activation of thrombocytes will eventually lead to the formation of a blood clot between the implant and the surrounding tissue [55]. Furthermore, according to Gittens et al., the impact of implant surface wettability extends beyond its involvement at the protein and cellular levels, as demonstrated in vivo and in the clinic [6].

A positive relationship between high surface roughness and surface hydrophilicity was also discovered by the studies of Bagambisa et al. [56], Buser et al. [57], and Bowers et al. [58]. Increased bone-to-implant interactions were seen for clinically relevant, superhydrophilic modified blasted and acid-etched surfaces compared with machined surfaces and hydrophobic blasted and acid-etched controls in vivo implantations. It is conceivable that faster, more reliable osseointegration might increase implant coverage for patients with compromised health, improve the long-term health of the implant site, and even increase the lifespan of the implant, which is challenging to assess in vitro, in vivo, or even in the clinic.

Scarano et al. [59] conducted a study with fresh non-heparinized blood or with other anticoagulant medication and a type of platelet concentrate as the wetting fluid for an evaluation that mimicked clinical reality. They stated that, as most of the studies that are present in the literature did not use blood as a wetting liquid, their studies could not demonstrate the role of the cells and proteins in the blood that can influence the viscidness of the fluid. As a result of their study, a statistically significant difference in wettability was found between the two types of surface and lower contact angle values on the sandblasted and double-acid-etched surface. In light of the results obtained, it is possible to affirm that the wettability and the interaction of autologous platelet liquid and blood assessed with a static method, despite the different cellular composition, look similar on rough surfaces; therefore, the static contact angle values seem to be influenced more by the surface characteristics than by the rheological properties of the wetting fluid. Our results were also in accordance with the findings of the studies that were mentioned above, as no statistically significant difference was found between the contact angle measurements of the groups. There was a statistically significant difference in RBC, HGB, and PLT values between the BTM patients’ and non-BTM patients’ blood samples; however, no statistically significant difference was found regarding their contact angles on Grade 5 implant discs that were blasted with biphasic calcium phosphate micro-blast with 65% HA content.

This study had an unpredictable major limitation. In the initial phase of our study, we requested different implant discs from different companies in order to also compare the implant technologies with each other. However, following the start of the study, the SARS-CoV-2 lockdown occurred to control its spread, where most countries enforced significant constraints that adversely affected the logistics and transportation sectors. Although we reached an agreement with some of the companies, they apologetically informed us that it will be impossible to provide the discs because of the prohibitions. As a consequence, we were only able to receive implant discs from a single company; thus, we could not perform our study with various discs.

In the wake of the results reported by Koca et al., this study was designed to see whether the differences between hyperlipidemic and healthy individuals were applicable to BTM patients and healthy individuals. Their study stated that, regardless of the surface, increased cholesterol levels cause decreased surface energy and wettability on the implant surfaces [13]. Fortunately, no statistically significant difference was found between the BTM patients, BT carriers, and healthy patients’ wettability features for theta-circular, theta-ellipse, and theta-mean values. As there was no statistically significant difference between all three contact angle measurements between the groups, this paper does not discuss whether one of these contact angle measurement methods is more significant. In order to improve the results and clinical appliances of this in vitro study, further prospective studies should be conducted to assess the implant success and osseointegration rates in BTM patients with different implant surfaces.

## 5. Conclusions

BT patients suffer from late wound healing, which is mainly attributed to low hemoglobin reducing tissue oxygenation, decreasing deformability, and increasing agreeability of erythrocytes attributed to high platelet levels that negatively affect perfusion. In order to detect whether this condition will affect the early phase of implant treatments, a contact angle measurement comparison was performed between BTM patients and healthy individuals; however, none of the measurements showed any significant difference between the groups, which suggested that blood samples of all groups had similar wettability properties in this in vitro study (*p* > 0.05). Hence, it is fair to state that while some systemic diseases may affect the contact angle between the implant surface and blood, it can be concluded that this condition was not present for the BTM patients in our in vitro study model. Prospective clinical studies must be performed to understand whether this result is practicable, and the same methodology should be applied to all compromised patients with systemic diseases. Last but not least, we emphasize that this experiment was done on a single surface type and the results can be totally different when using other surface types.

## Figures and Tables

**Table 1:** Demographic data of the patients.

Demographic Data of the Participants	Mean Age (Year)	Min Age (Year)	Max Age (Year)	Number of Female Participants	Number of Male Participants
Healthy participants	35.85	21	66	22	20
BT carriers	37.3	22	64	23	19
BTM patients	39.7	20	55	22	20

**Table 2:** Mean values with minimum and maximum values for RBC, HGB, and PLT values of the participants.

	RBC	HGB	PLT
	(Million Cells/mcL)	(gr/dL)	(Thousand Platelets/mcL)
Healthy male and BT carrier male	5.52 (min 4.72/max 6.06)	15.3 (min 13.45/max 16.41)	324 (min 174/max 445)
BTM male	3.76 (min 3.19/max 4.34)	9.91 (min 9.00/max 11.40)	525 (min 190/max 917)
Healthy female and BT carrier female	4.83 (min 4.23/max 5.26)	13.53 (min 12.1/max 14.83)	317 (min 162/max 434)
BTM female	3.79 (min 3.15/max 4.29)	10.09 (min 8.70/max 11.30)	470 (min 211/max 754)

**Table 3:** Comparison of the statistical analysis results of the blood sample features between the groups (* Mann–Whitney U test, ** Student’s *t*-test).

Parameters	Comparison Groups	*p*-Value
	BTM male vs. non-BTM male	0.000 *
	BTM female vs. non-BTM female	0.000 *
RBC	BTM male vs. BTM female	0.751 **
	Non-BTM male vs. non-BTM female	0.000 *
	BTM male vs. non-BTM male	0.000 *
	BTM female vs. non-BTM female	0.000 *
HGB	BTM male vs. BTM female	0.416 **
	Non-BTM male vs. non-BTM female	0.000 *
	BTM male vs. non-BTM male	0.002 *
	BTM female vs. non-BTM female	0.010 *
PLT	BTM male vs. BTM female	0.338 **
	Non-BTM male vs. non-BTM female	0.319 *

**Table 4:** Angular measurements, standard deviation, and uncertainty results.

	Theta-Circular	Theta-Ellipse	Theta-Mean
	(Degree)	(Degree)	(Degree)
Healthy participants	89.89	87.6	87.59
BT carriers	88.02	85.77	85.79
BTM patients	93.95	89.98	89.98
Distilled water	103.8	102	103.03
Isotonic water	102.7	101.3	102.14

**Table 5:** Statistical analysis of theta-mean, theta-circular, and theta-ellipse values.

	Theta-Mean (Degree)	Theta-Circular (Degree)	Theta-Ellipse (Degree)
Kruskal–Wallis H	1.227	2.926	1.226
Degrees of freedom	2	2	2
*p*-value	0.541	0.232	0.542

## Data Availability

The data sets used and/or analyzed during the current study are available from the corresponding author upon reasonable request.
